# Minimal resin embedding of SBF-SEM samples reduces charging and facilitates finding a surface-linked region of interest

**DOI:** 10.1186/s12983-023-00507-x

**Published:** 2023-08-29

**Authors:** Barbora Konopová, Jiří Týč

**Affiliations:** 1 Institute of Entomology, Biology Centre CAS, České Budějovice, Czech Republic; 2 Department of Zoology, Faculty of Science, University of South Bohemia, České Budějovice, Czech Republic; 3 Institute of Parasitology, Biology Centre CAS, České Budějovice, Czech Republic

**Keywords:** 3D imaging, Arthropod, High resolution, Optical sectioning, ROI localization, Serial block face, Volume EM, SBEM, Specimen charging, Sub-slice imaging

## Abstract

**Background:**

For decoding the mechanism of how cells and organs function information on their ultrastructure is essential. High-resolution 3D imaging has revolutionized morphology. Serial block face scanning electron microscopy (SBF-SEM) offers non-laborious, automated imaging in 3D of up to ~ 1 mm^3^ large biological objects at nanometer-scale resolution. For many samples there are obstacles. Quality imaging is often hampered by charging effects, which originate in the nonconductive resin used for embedding. Especially, if the imaged region of interest (ROI) includes the surface of the sample and neighbours the empty resin, which insulates the object. This extra resin also obscures the sample’s morphology, thus making navigation to the ROI difficult.

**Results:**

Using the example of small arthropods and a fish roe we describe a workflow to prepare samples for SBF-SEM using the minimal resin (MR) embedding method. We show that for imaging of surface structures this simple approach conveniently tackles and solves both of the two major problems—charging and ROI localization—that complicate imaging of SBF-SEM samples embedded in an excess of overlying resin. As the surface ROI is not masked by the resin, samples can be precisely trimmed before they are placed into the imaging chamber. The initial approaching step is fast and easy. No extra trimming inside the microscope is necessary. Importantly, charging is absent or greatly reduced meaning that imaging can be accomplished under good vacuum conditions, typically at the optimal high vacuum. This leads to better resolution, better signal to noise ratio, and faster image acquisition.

**Conclusions:**

In MR embedded samples charging is minimized and ROI easily targeted. MR embedding does not require any special equipment or skills. It saves effort, microscope time and eventually leads to high quality data. Studies on surface-linked ROIs, or any samples normally surrounded by the excess of resin, would benefit from adopting the technique.

**Supplementary Information:**

The online version contains supplementary material available at 10.1186/s12983-023-00507-x.

## Background

A current boom in volume electron microscopy has promoted serial block face scanning electron microscopy (SBF-SEM; also known as SBEM) as an ideal tool for imaging objects as large as 1.5 × 1.5 × 0.7 mm^3^ [26, 35, 36, 47]. With its high resolution it enables the study of individual organelles and their compartments in whole isolated cells, dissected organs and even entire bodies of small invertebrates (examples in [10, 14, 35, 43, 48, 49, 51]). Volume SEM provides invaluable information such as precise quantification of imaged data [14] and the detection of spatial connections and relations among the tissues/cells that would be otherwise missed by standard transmission electron microscopy (TEM), in which only a few selected sections from the studied tissue are observed in the microscope. Serial section TEM (ss TEM; i.e. imaging of each individual section that had been cut manually) has advanced this approach and provided critical insight into (sub)cellular structures [28, 37, 42, 52]. However, ssTEM is time consuming and requires unprecedented manual and technical skills (see also e.g., [36] for comparison with volume SEM). SFB-SEM is being used not only by cell biologists but also by other scientists, including zoologists, for whom it might open a whole new realm of information (e.g., [3, 5, 16, 18, 19, 21, 25, 40, 48, 49]). SBF-SEM has the potential to maximize the information contained in any sample volume, be it an animal, plant or protist, at high resolution.

SBF-SEM is a scanning electron microscope (SEM) with a built-in diamond knife ultramicrotome that repeatedly removes sections from a resin embedded sample [8, 24, 47]. The surface is scanned after each cut and stacks of TEM-like images are obtained with backscattered electrons (BSE) and combined with image processing software. Because sectioning and image acquisition are highly automated, SBF-SEM offers the unprecedented opportunity to obtain a large volume of information with relative ease.

Despite SBF-SEM being a robust technique, it suffers from specific problems (e.g., [26, 36, 47]). These relate particularly to the fact that the tissue is (as standard) buried in a mass of embedding medium, the resin, which is poorly conductive. Firstly, frequent specimen surface charging leads to poor image quality and lower resolution. Secondly, because the sample is obscured by overlying resin, the region of interest (ROI; the specific part of the sample, which is a subject of the research project) is difficult to find. Trimming the sample in the imaging chamber and navigating the microscope to the ROI is then complicated.

These problems are especially relevant, if the imaged region includes the surface of the sample. This happens in many cases, e.g., when whole chunks of small animals are embedded to image a certain appendage, sensilla or cells associated with a specific surface microstructure. SBF-SEM studies on arthropods provide an example [3, 15, 19, 21, 25]. These samples are especially sensitive to charging, as explained below. Also, the advantage that their ROI could easily be localised from cues in external morphology (in contrast to ROIs within a piece of tissue cut from a large organ, such as the mouse brain) is lost, because the surface is masked by the external resin. In this study, we focus on such samples with surface-linked ROIs.

Charging is one of the biggest limitations of SBF-SEM [8, 47]. It is manifested as contrast abnormalities, such as dark streaks and regions, and image deformation [7, 8, 33]. Charging occurs when the electron beam interacts with the nonconductive resin in which the sample is embedded (e.g., [8]). Under high vacuum conditions some electrons’ energy cannot dissipate to earth and charge accumulates [7, 30]. Although biological objects are also non-conductive, they are impregnated and stained with heavy metals, which improves conductivity. Charging is therefore more pronounced in regions containing the empty resin, which can be found first of all around the sample.

Several strategies are being employed to deal with the charging artefacts. The easiest solution is to image in the low-vacuum mode (also known as residual-gas method or variable-pressure mode) [8]. This, however, leads to a decrease in resolution (less detailed, more noisy images) compared to those obtained in the optimal high-vacuum mode. Devices built into the microscope chamber, which modify the imaging conditions, have provided solutions in several cases, but they have other technical limitations [46] or they are not compatible with some microscopes due to patents [7] and thus they are not available to everyone. Improvements in the sample preparation methodology may help [9, 34, 39]. Surrounding the sample with a conductive medium [32, 50], prevents charging, but these media are dark and a little opaque, thus hiding the sample further [7, 43]. In summary, most of the strategies are effective in some applications, but not suitable for every sample and microscope.

Another limitation of SBF-SEM imaging is that it is a lengthy process and hence costly [47]. Frequently, several days to weeks are required to section and scan only the ROI. Additionally, significant time is spent on trimming the samples in the imaging chamber before imaging can even start. In samples embedded traditionally *en bloc* [8, 26, 39] even the surface linked ROI is difficult to find. The sample cannot be trimmed precisely to the desired starting point of imaging prior to being placed into the microscope. In addition, the ROI could be accidentally cut off. Trimming in the imaging chamber creates unwanted debris there and the diamond knife inevitably blunts. The extra time that the sample has to spent in the microscope raises the cost of the final dataset.

The minimal resin (MR) embedding method was developed to enable easy localization of the ROI on the surface of a sample in focused ion beam SEM microscopy (FIB-SEM) [41]. FIB-SEM also traditionally uses *en bloc* embedded samples [11, 22, 31]. Thin layers of material from the imaged area are subsequently removed by milling with a focused beam of ions. This restricts imaging to much smaller areas compared to SBF-SEM, but charging is typically not an issue [47]. According to the MR embedding protocol [41, 44], the specimen is infiltrated with a classical resin, but before it is polymerized (cured, hardened) the surrounding layer of resin is blotted away. This enables visualisation of fine landmarks in the external morphology using secondary electron (SE) imaging. The suitability of MR embedding for SBF-SEM has not yet been tested.

In this study we had two goals. Firstly, to develop a workflow for preparing samples for SBF-SEM using MR embedding and to determine if the samples could be reliably sectioned by the microscope microtome without the support of the external resin. Secondly, to test whether MR embedding of samples helps to reduce charging, so that these samples could be observed in high vacuum and more detailed images thus obtained. We chose two types of samples: (1) we embedded trunks of three small arthropods, a springtail *Orchesella cincta* (Collembola), a conehead *Acerentomon dispar* (Protura) and a two-pronged bristletail *Campodea franzi* (Diplura) to image specialized non-locomotory appendages on their first abdominal segment, and (2) we embedded a whole egg, a roe, of a fish *Oryzias latipes* (Adrianichthyidae) to be imaged in a full width from the top.

We show that our samples were stable and did not vibrate during sectioning and imaging, and charging was absent or greatly reduced. We conclude that MR embedding is a convenient method that generates high quality data sets.

## Results

### MR embedding of SBF-SEM samples

**Fig. 1:**
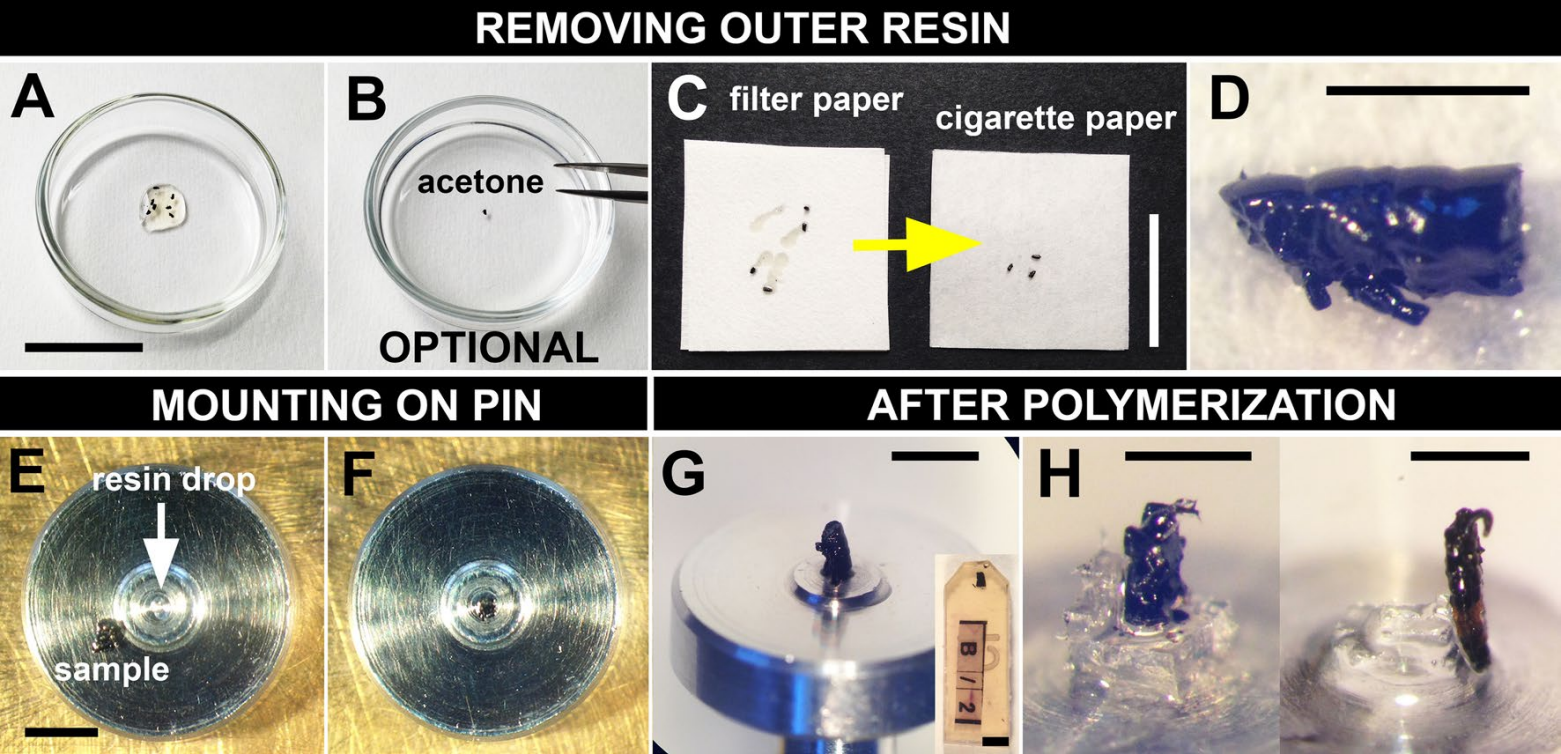
MR embedding for SBF-SEM. **A** Samples infiltrated with pure resin (Additional file 1) were poured into a glass petri dish to enable inspection of their integrity under the stereomicroscope. **B** Optional brief dipping in acetone. **C** Draining the outer resin. Samples (removed from acetone or directly from the resin) were transferred onto a piece of filter paper, moved around and then placed on cigarette paper. **D** Sample after removal of outer resin. **E** Mounting on a pin. A SBF-SEM pin was placed under the stereomicroscope and using a toothpick a small drop of resin was made in the centre. Using clean forceps the sample was placed on the side of the pin to see the position of the ROI. **F** Sample was placed on the drop in a desired orientation for imaging. **G** Sample on the pin after it was polymerized. Compare with the traditional embedding in a block (inset). **H** Minute sample polymerized on a resin pillar, which supports it from the bottom (left); elongated sample is supported from the side (right). Samples: **A**–**G** *O. cincta*, **H** *A. dispar*. Scale bars: **A**, **C**, 2 cm; **D**, 1 mm; **E**, **G**, inset in **G**, 2 mm; **H** left, 400 μm; **H** right, 500 μm

**Fig. 2:**
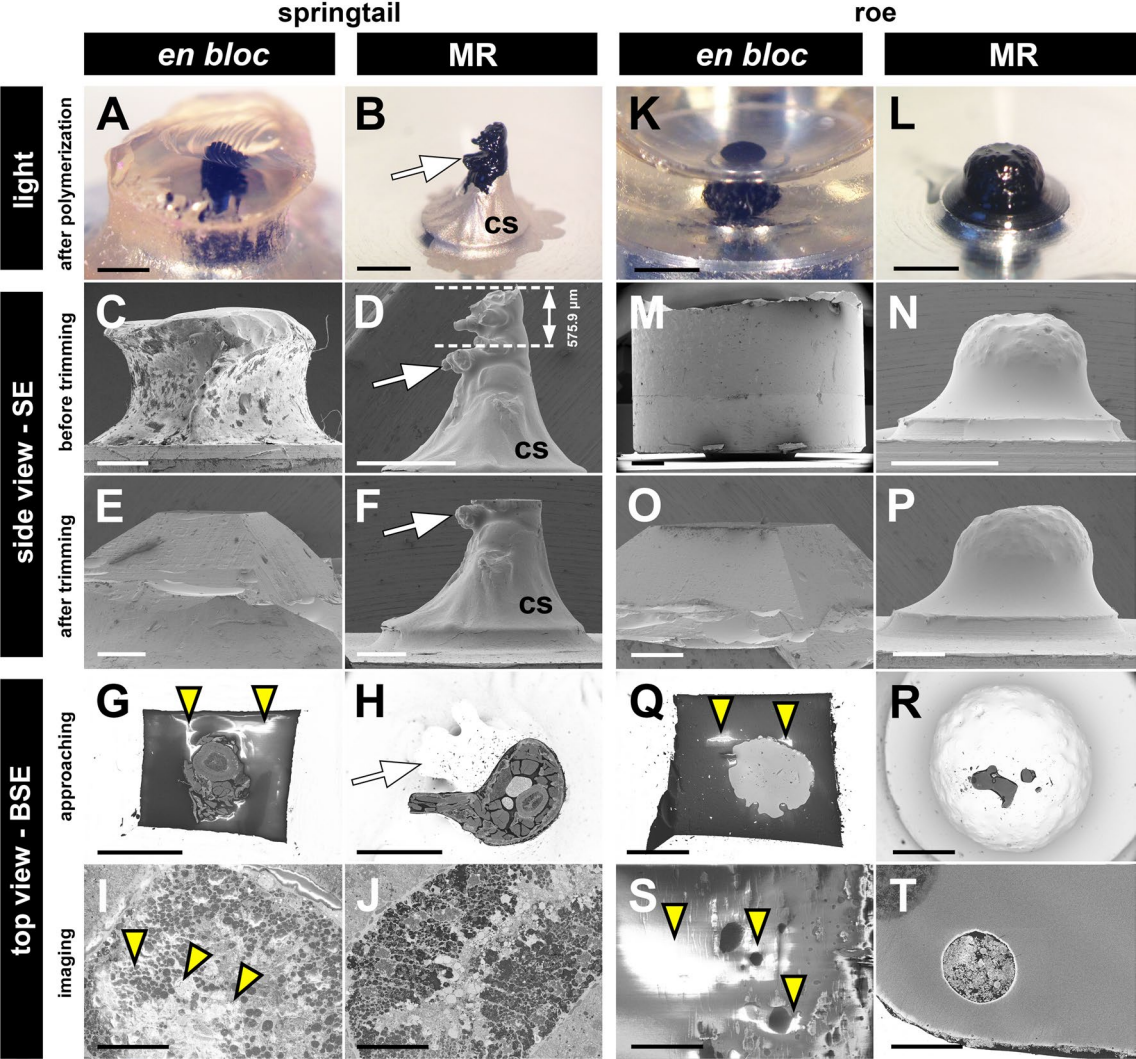
Trimming, approaching and sensitivity to charging in *en bloc* and MR embedded samples. **A**, **B**, **K**, **L** Polymerized samples on pins observed under the stereomicroscope before they were sputter gold coated. Colloidal silver (cs) was applied around the base of the sample in **B**. **C**–**F**, **M**–**P** Side views of samples visualized in the classical SEM mode using SE. **C**, **D**, **M**, **N** Measuring the length of the material above the ROI (white arrow) that has to be trimmed off. These values were estimated in *en bloc* embedded samples (**C**, **M**). **E**, **F**, **O**, **P** Trimmed samples. Only a few sections of outer resin were trimmed in **P**. A classical pyramid was made in **E** and **O**. **G**–**J**, **Q**–**T** Top views of samples visualized in the SBF-SEM mode using BSE. **G**, **H**, **Q**, **R** Overview during approaching. **I**, **J**, **S**, **T** Images of embedded tissue after a few sections were cut. All observed in high vacuum. Yellow arrowheads mark charging (white regions). Samples: **A**–**J** Trunk fragment of *O. cincta*; ROI: an appendicular organ (collophore) in the middle of the body. **K**–**T** Roe of *O. latipes*; ROI: full width from the top. cs, colloidal silver; white arrows mark the ROI; yellow arrowheads mark charging. Scale bars: **A**–**D**, **K**–**N**, 1 mm; **E**–**H**, **O**–**R**, 500 μm; **I**, **J**, **S**, **T**, 30 μm

To embed the samples we modified the protocol for FIB-SEM [41, 44]. For details see Fig. 1 and Methods. The term “sample” is used here exclusively for the biological object. In parallel we prepared samples (springtails and roes) embedded in the classical manner inside a mass of resin (*en bloc*) for comparison (compare Fig. 2A, K with B, L).

Similar to the FIB-SEM protocol [41, 44], we blotted away external resin on the samples (Fig. 1A) by placing them on absorbent paper (Fig. 1C). To facilitate removal of the resin we added an optional step, where the samples were first dipped for a few seconds into acetone before they were placed on the paper (Fig. 1B). This was useful for samples where resin had to be removed from small crevices and/or where detailed ultrastructural landmarks were necessary (Additional file 2).

Samples stripped of the external resin (Fig. 1D) were positioned in the desired orientation for imaging on the SBF-SEM pin and attached to it using a drop of resin as a “glue” (Fig. 1E–G). Where possible the samples were so positioned that the ROI was not at the top, but some part of the object projected above it (Additional file 3: A). Having the small amount of extra material above the ROI was convenient for the alignment of the knife and the initial approaching steps in the SBF-SEM microscope (see Methods: Trimming of samples). The amount of resin to attach the sample had to be small. If too much was applied it rose up by capillary action and could mask the surface including the ROI (Additional file 4: A). Samples attached with little contact to the pin could vibrate during sectioning (e.g., tall samples), therefore colloidal silver was applied around the base to secure them more robustly. The base must be firmly attached to the pin and should be broader than the top of the sample.

In samples so small that the ROI would be too close to the pin (< ~ 200 μm) (Additional file 4: B), there is a risk that the diamond knife hits the metal pin and loses its edge. Therefore we in advance prepared pins with a support pillar, on which the samples were placed (Fig. 1H, left). A specifically shaped pillars also served as a support for elongated samples that had to stand upright (Fig. 1H, right).

### Surface ROIs are easy to find and navigate to

Without the extra resin, the whole MR embedded sample could easily be seen by the naked eye, under the light stereomicroscope, as well as in the SEM (Fig. 1G, H, 2B, D, F, L, N, P, Additional file 5: B, D, D', F, F'; compare with the *en bloc* embedding in Fig. 1G inset, Fig. 2A, C, E, K, M, O, Additional file 5: A, C, C', E, E'). Side view SEM images were used to measure precisely the extent of the material that required trimming off (Fig. 2D, Additional file 5: D; compare with *en bloc* embedding shown in Fig. 2C, M, Additional file 5: C, C'). This was carried out using a lab ultramicrotome (all samples after trimming are shown in Fig. 2E, F, O, P, Additional file 5: E–F′). It was easy to navigate the SBF-SEM microscope to the ROI, because the whole depth of the sample could be seen (Fig. 2H, Additional file 3: B and 5: H). In the case of the *en bloc* embedded samples one orients itself from the top view images (Fig. 2G, Q, Additional file 5: G).

### Less resin around the sample results in less charging

To compare the occurrence of charging in the SBF-SEM mode between *en bloc* and MR embedded samples we scanned three pairs of these prepared in parallel using each method in high vacuum (panels “top view—BSE” in Fig. 2 and Additional file 5; imaging conditions are in Additional file 6). A few (3–5) rounds of sectioning-scanning were completed for each sample. While all *en bloc* embedded samples showed some level of charging (similarly as other diverse *en bloc* embedded samples in our laboratory, data not shown) which obscured the ultrastructural information (Fig. 2G, I, Q, S, Additional file 5: G, I, I′). By contrast, charging in the MR embedded samples was minimal to absent (Fig. 2H, J, R, T, Additional file 5: H, J, J′). If it occurred it was in the residual resin remaining around the sample or in the empty resin filling up cavities inside of it. Importantly, it did not obscure cellular ultrastructure (Additional file 5: J′).

### MR embedded samples are stable during the SBF-SEM run

**Fig. 3:**
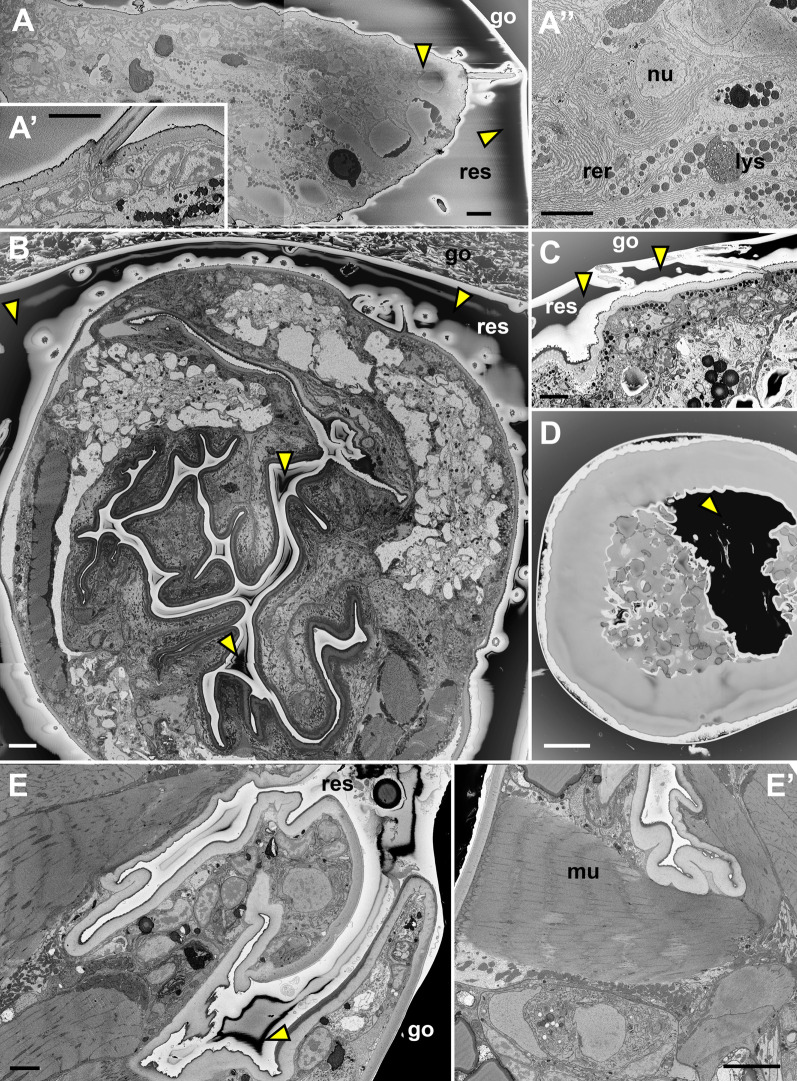
Examples of SBF-SEM images acquired from MR embedded samples. Single scans of the sample surface, several tiles stitched together. **A**–**A″** Less successfully embedded sample, where too much resin (res) around it remained. Low vacuum mode, 20 Pa. **B**–**E**′ Resin sufficiently removed. High vacuum mode. Samples (details in Additional file 6): **A**–**A″** *C. franzi*—sample 6 (first abdominal appendage—longitudinal section); **B** *O. cincta—*sample 1 (collophore—cross section); **C** *O. cincta*—sample 2 (trunk body wall); **D** *O. latipes* (whole roe); **E**, **E**′ *A. dispar*—sample 5 (proximal part of the first abdominal appendage, neighboring muscles). Arrowheads mark residual charging around the sample (black regions in these inverted images). go, gold sputter coating; lys, lysosome; mu, muscle; nu, nucleus; rer, rough endoplasmic reticulum; res, resin. Scale bars: **A**–**A″**, **C**–**E′**, 5 μm; **B**, 10 μm

**Fig. 4:**
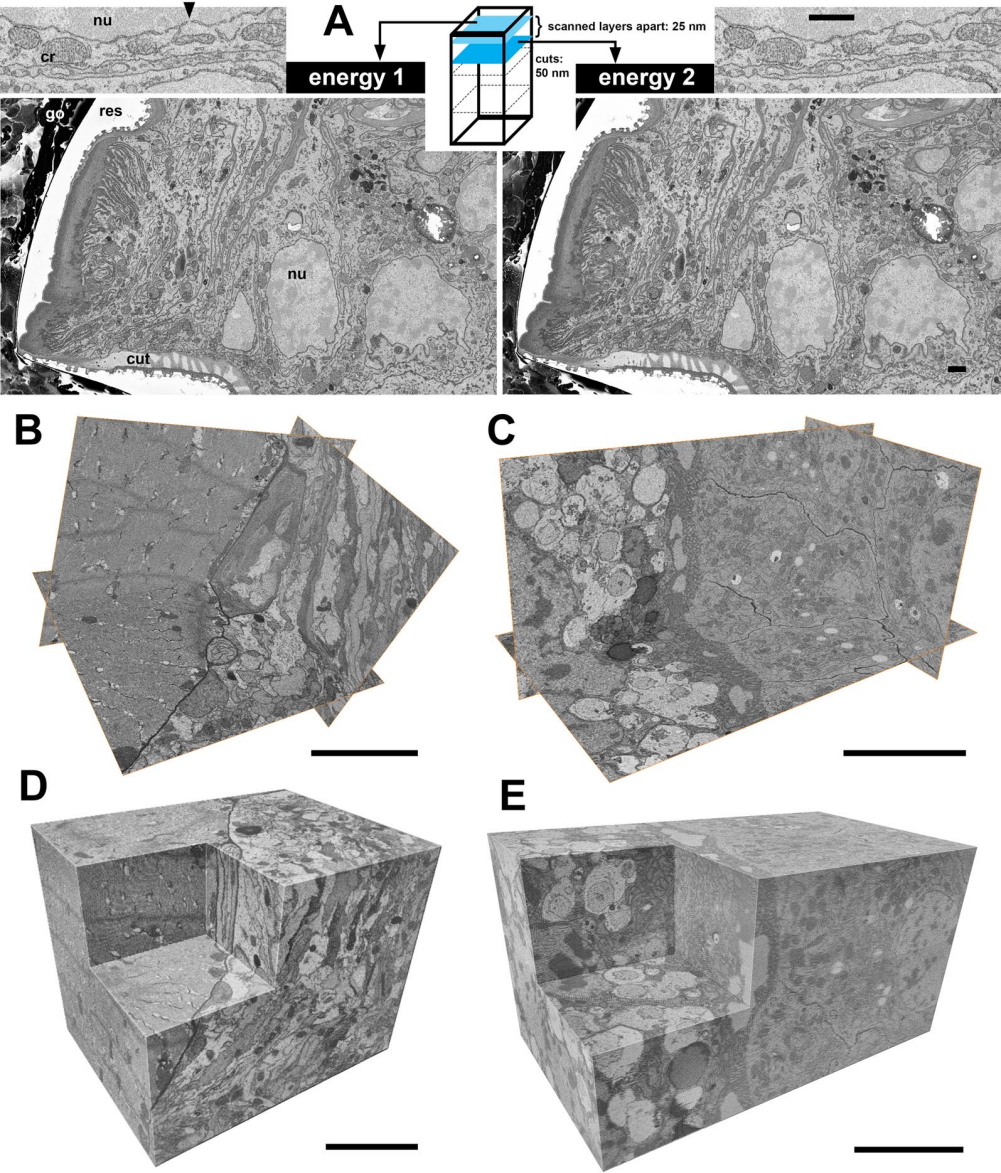
Sub-slice imaging (optical sectioning). **A** Layers 50 nm thick were cut using a diamond knife and two primary beam energies (2.5 kV and 4 kV) were used to scan information from layers 25 nm apart (scheme). Single scans of cytoplasm and mitochondria in a transporting epithelium (images). Volume data from this area are in the movie in Additional file 8. **B**–**E** High-resolution trial datasets. Volume reconstruction of muscle and adjacent tissue (**B**, **D**) and gut tissue (**C**, **E**) showing cross-sections in all three axes (**B**, **C**) and volume rendering (**D**, **E**). Data was acquired by a combination of physical (40 nm) and virtual (10 nm) slicing. The voxel size was 8 × 8 × 10 nm^3^ with a volume of 16 × 12 × 13 μm^3^ (**B**, **D**) and isometric voxel 10 × 10 × 10 nm^3^ with a volume of 23 × 13 × 12 μm ^3^ (**D**, **E**). Movies for **B**, **D** and **C**, **E** are in Additional files 9 and 10, respectively. Samples: **A** *O. cincta*—sample 1, **B**, **C** *A. dispar*—sample 3, **D**, **E** *O. cincta*—sample 6. (Additional file 6). cr, cristae in mitochondria; nu, nucleus; res, resin. Arrowhead marks a nucleopore. Scale bars: 5 μm

To verify the stability of the conditions during image acquisition in 3D, we performed several proper SBF-SEM runs for volume imaging. In total we imaged six arthropod samples and seven samples of fish roe (Figs. 3, 4, list of the samples and imaging conditions are in Additional file 6).

All arthropod samples, in which the surrounding resin was sufficiently removed (Fig. 3B–E′), were observed in high vacuum. We used short pixel dwell times (time needed for scanning one pixel), ~ 0.3–1.2 μs, and low electron dose, ~ 5–16 eV/nm^2^ (Additional file 6). Note that for samples suffering charging, so that they have to be imaged in low vacuum, these values may exceed 5 μs and 35 eV/nm^2^, respectively, but thicker sections generally have to be cut (see Discussion). Additional file 7 documents that detailed, high resolution images were obtained. Although most of our samples were sectioned at 100 nm in order to acquire data from a large volume in a reasonable time, we were able to cut the arthropod MR embedded samples at 50 or 40 nm without difficulties (Additional file 6).

In one of the samples we did not remove the resin properly (Fig. 3A–A″, sample 6 in Additional file 6). A thicker non-conductive layer then remained between the sample and the gold sputter coating (a standard coating for SEM samples to increase the conductivity of the surface, see Methods), as seen on SBF-SEM images (“res” in Fig. 3A). This sample suffered from charging and we had to observe it in the low vacuum mode. Because the charging was not excessive, the relatively good (relatively “high”) vacuum (Additional file 6) could be used and detailed images were still obtained.

### High resolution z-imaging is possible

To increase the z-resolution we applied sub-slice imaging aka optical sectioning mode [2, 6]. This is possible in the Thermo Fisher Scientific microscopes using the imaging and the processing software MAPS [45], but requires samples that can be scanned with low electron doses and under good vacuum conditions. In sub-slice imaging [2, 6] multiple primary beam energies are applied to collect information from different depths of the sample after each cut. We cut 50 nm sections using a diamond knife and then applied two primary beam energies (2.5 kV and 4 kV) to scan information from layers 25 nm apart (Fig. 4A, movie in Additional file 8). We obtained a 3D dataset at voxel size of 8.5 × 8.5 × 25 nm^3^. In pilot experiments on a smaller volume, which were performed specifically to find the potential imaging limits, we were able to get to 40 nm physically cut sections. Using sub-slice imaging the final achieved voxel size was 10 × 10 × 10 nm^3^ (isometric) and 8 × 8 × 10 nm^3^, respectively (Fig. 4B–E, movies in Additional files 9 and 10). This further supports the benefit of MR embedding in reducing the limitations for very thin sectioning and high-resolution imaging.

## Discussion

### MR embedding helps with two problems in SBF-SEM simultaneously

On the examples of surface-linked ROIs we have successfully tested the MR embedding method for tackling two major problems of SBF-SEM: charging and finding the ROI. Our workflow for MR embedding stems from the protocol developed for finding surface ROIs in FIB-SEM [41, 44]. While in FIB-SEM the main purpose was to localize and image the surface-linked ROIs, we show that in SBF-SEM the MR embedding has another big advantage and that is charging reduction.

### Samples prepared according to our MR embedding protocol could be reliably sectioned by the microscope microtome

We made a few modifications to the original protocol. To facilitate the removal of the external resin we added an optional step, in which we briefly dipped the samples into acetone. Steyer et al. [44] used a halogen lamp to make the resin less viscous. Acetone dissolves the resin and the samples cannot stay in it for too long. But the resin is removed fast even from small crevices on the surface of the sample, while the inner parts still remain well infiltrated. We polymerized the samples directly on the SBF-SEM pins attached with a drop of resin. To secure the samples that might not attach enough and to prevent their vibration during cutting, colloidal silver was applied around the base. It does not have so strong capillary action and stays at the bottom. It also contributes to the conductivity of the surface (see below). In the FIB-SEM protocol samples were first polymerized onto a plastic film and only after than attached to the pins using either conductive carbon sticker and silver paint (= colloidal silver liquid) [41], or silver resin (= EPO-TEK EE 129–4) and polymerized again [44].

We did not encounter any problems during the SBF-SEM run, such as extensive chatter, irregular thickness of sections during cutting (manifested as imaging the same section twice—no cutting, followed by a subsequent thicker section) or artefacts that could originate from sample instability. However, it was important to ensure that the base of the sample was firmly attached to the SEM pin and was broader at the point of attachment than at the top of the object. For thin samples resin pillars were used as a support (Fig. 1H).

### MR embedded samples are easy to set up for imaging

As in FIB-SEM the great advantage of MR embedding is the easy navigation to the surface ROI. In SBF-SEM this happens during trimming. The extra material above the ROI can be precisely and quickly trimmed off before the samples are placed into the microscope. Because the ROI can be clearly seen under the stereomicroscope, the risk that it is accidentally trimmed off is minimized. Inside the SBF-SEM microscope, it is easy to find one’s way around an MR embedded sample because the whole object is visible (Fig. 2H, R, Additional file 3: B and 5: H). Navigating the microscope to the ROI is then fast. No extra trimming is required. From our experience with other samples processed in our facility we estimate that a couple of hours to days can be saved on the initial approaching step in the SBF-SEM microscope. At facilities that charge for imaging time, this might save the user several hundreds EUR for obtaining the dataset. For ROIs that are not on the surface or associated with it (such as an organ in which a particular cell is targeted) conventional extensive trimming in the imaging chamber has to be used. The alternatives for some of these samples may be e.g., X-ray microscopy [27]. We do not address the tissue samples and internal ROIs here and focus on surface-linked ROIs for which the MR embedding is the easiest solution.

### MR embedding efficiently reduces charging

In our hands, the huge improvement with the MR embedding was the reduction of charging. We showed that the mere removal of resin around the sample using MR embedding, if done properly, reduces image-destructive charging to the degree that imaging could take place in high vacuum. This enables to obtain high resolution images in which ultrastructural details are observed (Fig. 3, 4, Additional file 7). It is therefore not necessary to coat samples with conductive alternatives [32, 50], which obscure the sample’s morphology. A thin layer of gold sputter coating, a standard for SBF-SEM samples, is sufficient. The colloidal silver at the base is not critical for charging reduction, as we were able to observe certain samples in high vacuum without it—even the sample with the final voxel size 8.5 × 8.5 × 25 nm^3^ (Fig. 3B, Fig. 4A, Additional file 3: A, Additional file 7: A, B, D, G, J, Additional file 8). The layer of colloidal silver is a help for curved samples, in which sputter coating cannot cover the crevices at the base completely. These less conductive spots may contribute to charging artefacts. For the conductivity of the surface of the sample it is also important that the base is firmly connected to the pin. Our simultaneously prepared *en bloc* embedded samples suffered charging so that they would need to be observed in low vacuum. Even residual charging is problematic, as it hampers alignment in 3D reconstructions [8] and generally complicates image processing. Imaging in high vacuum without charging then results in better resolution, less noise and lower electron dose.

For reliable sectioning in SBF-SEM it is recommended not to exceed the electron dose limit 19–25 eV/nm^2^. Nevertheless higher dose is sometimes necessary. Especially, if electrons are scattered in low vacuum and only a fraction is detected [17, 29]. For example, in our previous study on single cell diplonemids where the extra resin could not be avoided [38], and high details were required, electron dose 34.7 eV/nm^2^ and (long) pixel dwell times 4 μs had to be used. A final pixel size 6 × 6 nm^2^ in x, y was achieved, but the voxel thickness in z had to be sacrificed and 100 nm thick sections had to be cut to ensure reliable cutting. Comparable parameters, including long dwell times and thicker sectioning were used in similar studies (e.g., [12, 20]).

The low electron doses that we could use for imaging the MR embedded samples, ~ 5–16 eV/nm^2^, and therefore reduced beam damage enabled us to perform sub-slice imaging [2, 6] (Fig. 4). Low electron doses are required, because the final dose is a sum of the distinct energies used. With 50 nm physical sectioning and two primary beam energies applied we achieved 25 nm voxel thickness in z. Our voxel size 8.5 × 8.5 × 25 nm^3^ was then comparable to that achieved by Wanner et al. [50], 9 × 9 × 25 nm^3^, who used silver epoxy embedding to prevent charging and were able to section physical slices of 25 nm. In our pilot experiment on a smaller volume, by which we wanted to test the limits of our successfully MR embedded samples, we managed to reliably cut 40 nm layers and with the 10 nm sub-slice imaging we reached the isometric voxel size 10 × 10 × 10 nm^3^ and in another dataset 8 × 8 × 10 nm^3^ (Fig. 4B–E, Additional files 9 and 10). This voxel size is even comparable to FIB-SEM [11, 22, 31] and on the very limit of SBF-SEM.

### Limitations and outlook

SBF-SEM was developed for imaging pieces of dissected tissue, such as brain and muscle [8]. The sample preparation logically stemmed from the protocol for TEM and embedding into a block of resin. Samples for SBF-SEM are generally much more variable in their nature. Our data show that for certain types of sample, the problematic external resin is not necessary and can be avoided. Samples with surface-linked ROIs can benefit even more due to easier navigation to the ROI.

There are limitations. MR embedding cannot be used for samples typically harvested as pellets such as unicellular organisms or isolated cells. It cannot prevent charging originating inside of tissues with large internal cavities filled with empty resin, such as lungs, blood vessels or vacuoles, tissues that are low in lipids or otherwise difficult to stain with heavy metals [26].

It is worth noting that as a whole, charging depends on the overall conductivity of the sample including the quality of infiltration with heavy metals. Samples predominantly composed of low atomic number elements are often insulators. These include many biological objects. We stained our samples according to a standard protocol [13] with minor modification. Advances in staining methodology [9, 34, 39], promise further help from this direction.

## Conclusions

The data presented here show that preparing SBF-SEM samples according to MR embedding efficiently decreases charging and facilitates finding surface-linked ROIs. It saves time on setting up samples for imaging, and as a result speeds up their turnover in the SBF-SEM microscope. Because the method does not require any special equipment, chemicals or new skills, any SBF-SEM lab can start using it right away.

## Methods

### Sample origin and fixation

Arthropods: *O. cincta* (Collembola) was obtained from a laboratory colony [23], *A. dispar* (Protura) was extracted from soil collected near Holubov (South Bohemia) and *C. franzi* (Diplura) was collected near Boršov nad Vltavou (South Bohemia).

Animals were anesthetized by CO_2_ and immersed into a freshly made fixative (4% formaldehyde with 2.5% glutaraldehyde in 0.15 M cacodylate buffer), the front and rear parts of the body were cut off to facilitate penetration of the solution. They were fixed at 4 °C for 5–15 days (*A. dispar*: 5 days, *C. franzi*: 8 days, *O. cincta*, samples 1 and 4: 15 days, samples 2 and 3: 5 days). Because the cuticle of *O. cincta* is hydrophobic and the animals float on the surface of the fixative, the individuals were kept submerged by being pinned with minutia pins to the bottom of a Sylgard-coated dish and later on pins transferred into tubes.

Fish roe: The eggs (roes) of *O. latipes* (Adrianichthyidae) were kindly provided by Martin Pšenička. The fixative was as above. Because in the initial runs we had problems with the resin penetration through the egg envelopes (chorion), we settled on a protocol where the chorion was pierced with needles at the beginning of fixation. Additionally, for all steps the samples were also centrifuged for 10 min at 1000 G and microwaved at water bath in a regular kitchen microwave (Miele) at the lowest power (80 W) for 60 s (a plastic container was filled with water, samples were placed into a foam or polystyrene tube rack floating on the water surface, the whole container with water and samples were transferred into the microwave) to ensure proper penetration and exchange of solutions.

### Heavy metal staining and resin infiltration

Immediately after fixation, samples were stained with heavy metals and infiltrated with epoxy resin according to a standard protocol for SBF-SEM [13] with modifications, details are in Additional file 1. Roe samples were in each step centrifuged and microwaved as above.

### MR embedding (additional notes)

Samples were handled using fine forceps, toothpicks and needles. For the optional acetone step, samples were dipped into a dish with 100% acetone or they were left there for several seconds and swirled a little. This was carried out with coneheads and the majority of springtails. Samples on SBF-SEM pins were polymerized at 60 °C for 48 h. Colloidal silver (Colloidal Silver Liquid, Electron Microscopy Sciences, Cat 12630) was diluted to a semi-viscous consistency according to the manufacturer’s protocol and applied around the base of the samples using a toothpick. This step was omitted in samples that were sufficiently attached with resin.

### Preparing pins with a support pillar

Using an ultramicrotome (Leica EM UCT7) and a glass knife, a protruding cube was cut at the tip of a regular resin block. A front half was cut to the middle of the cube to create the shape of “a chair”. The piece was cut off and attached to a pin with a drop of resin. The pin with the support pillar was polymerized.

Samples (long objects that had to stand upright and small objects where ROI would be located too close to the pin) were placed on these pins similarly as the other samples were placed on the pins without pillars: i.e. samples were transferred using fine forceps, positioned in the correct orientation on the pillar, to which they were attached with a small amount of resin and then the whole pin was incubated at the polymerization temperature.

### Trimming of samples

Samples, both *en bloc* and MR embedded (Fig. 2A, B, K, L and Additional file 5: A, B), were sputter coated with gold (direct sputter coating with rotation of the sample at an angle to create a 40 nm layer; Leica EM ACE200) and observed at a 90° angle in the SEM Jeol 7400 or Apreo SEM (Thermo Fisher Scientific) microscopes using SE (Fig. 2C, D, M, N, Additional file 5: C, C', D, D').

MR embedded samples: Distances between the highest point of the sample and the desired imaging start point were measured using the associated software (Fig. 2D, Additional file 3: A, Additional file 5: D). The top of the sample of the measured length minus ~ 10–20 μm (where possible) was cut off using a standard ultramicrotome in the lab: section thickness was set and the number of sections counted (Fig. 2F and Additional file 5: F, F′). Reaching the desired point was also checked visually. The extra material above the ROI was left for the alignment of the knife and initial approaching steps in the SBF-SEM. During the approaching step some sections are being cut and therefore lost. For the fish roe, only the initial couple of sections were trimmed off to obtain a smooth surface against which the knife in the SBF-SEM could be aligned (Fig. 2R).

*En bloc* embedded samples: Because the samples were masked by the external resin and could not be seen in SEM, the amount of material to be removed from the top was only roughly estimated from observations under the stereomicroscope using light (Fig. 2A, C, K, M and Additional file 5: A, C, C′). Standard pyramids were trimmed using a lab ultramicrotome and sputter coated (Fig. 2E, O and Additional file 5: E, E′). After being inserted into the SBF-SEM microscope the samples were trimmed to the desired imaging start point. The position of the ROI was (as standard) estimated from 2D images of the block surface (Fig. 2G, Q, Additional file 5: G).

### SBF-SEM, sub-slice imaging and image analysis

Samples were observed in Apreo SEM equipped with volumescope and variable pressure control (Thermo Fisher Scientific). Imaging conditions are summarized in Additional file 6. One or multiple (two or four) beam energies were used for standard and sub-slice imaging, respectively (details in Additional file 6). The acquisition of images and their deconvolution were carried out using the MAPS software (Thermo Fisher Scientific) with default parameters. The resulting datasets were processed and analysed using the softwares MAPS (Thermo Fisher Scientific), Microscopy Image Browser [1], TrakEM2 [4] and Amira (Thermo Fisher Scientific). Brightness and contrast in the Figures were adjusted using Adobe Photoshop CS6.

### Supplementary Information

**Additional file 1.** Protocol for heavy metal staining and resin infiltration used for samples in this study.

**Additional file 2:** Comparison of details in surface ultrastructure between samples prepared without and with the optional acetone washing of external resin. (A) The acetone-washing step omitted, (B) included. Samples: roe of *O. latipes*. Scale bars: 100 μm.

**Additional file 3:** Additional illustrations of the preparation of MR embedded samples for SBF-SEM imaging. (A) Measuring the sample for precise trimming. External resin on this sample was removed by draining on absorbent paper without the acetone step, attached to the pin only with resin (without colloidal silver). Note that a structure (leg stump) is protruding slightly above the level of the ROI, which enables knife alignment. SBF-SEM images from this sample are shown in Fig. 3B and Fig. 4A. (B) Navigation to the ROI in the SBF-SEM microscope. Images in (A) and (B) were obtained using SE. cs, colloidal silver; res, resin; ROIs are encircled. Samples: *O. cincta*. Scale bars: 100 μm.

**Additional file 4:** Incorrectly embedded samples. (A) Resin drop on the pin to attach the sample was too large. Resin rose up to the object (arrow). (B) Sample was very small, but mounted directly on the pin without the supporting pillar. Sectioning the ROI (asterisk) would take place too close to the pin (< ~200 μm). Samples: *C. franzi* (A) and *A. dispar* (B). Scale bars: 1 mm.

**Additional file 5:** Trimming, approaching and sensitivity to charging in additional *en bloc* and MR embedded samples. (A, B) Polymerized samples on pins before they were gold coated. Colloidal silver was applied around the base of the sample in (B). (C-F’) Samples visualized in the classical SEM mode using SE. (C, E, D, F) are side views, (C’, E’, D’, F’) are top views. (C-D’) Measuring the length of the material above the ROI that has to be trimmed off. These values were only estimated in the *en bloc* embedded sample (C). (E-F’) Trimmed samples. A classical pyramid was made in (E, E’). (G-J’) Top views of samples visualized in the SBF-SEM mode using BSE. (G, H) Approaching. The ROI is easily localized and navigation to it is uncomplicated in the MR embedded sample (H). In (G) it is not clear how distant the ROI is from the top of the pyramid. Charging (yellow arrowhead) is visible in the resin surrounding the *en bloc* embedded sample (G). (I-J’) Images of embedded tissue after a few sections were cut, both samples in high vacuum. Minimal charging appears in the empty resin inside the MR embedded sample, which does not obscure details in the cells. Massive charging, which spreads into the tissue, appears in the *en bloc* embedded sample (I, I’) and completely disrupts imaging. (I, J) are at lower and (I’, J’) at higher magnification. cs, colloidal silver; white arrows mark the ROI; yellow arrowheads mark charging. Samples: *O. cincta*. Scale bars: (A, B), 2 mm; (C, C’), 2 mm; (E’), 1mm; (D, D’, E, F, F’), 100 μm ; (G, H), 500 μm; (I, J), 10 μm ; (I’, J’), 50 μm .

**Additional file 6:** Imaging conditions.

**Additional file 7:** Ultrastructural details observed in SBF-SEM images of MR embedded samples. (A) Golgi complexes in the vicinity of the nucleus. (B) Mitochondria and the basal labyrinth (infolding of plasma membrane). Both (A, B), transporting epithelium in the collophore of *O. cincta*. (C) Gut cell, *O. cincta*. (D) Muscle, cross section. Collophore, *O. cincta*. (E) Muscle, cross section. (F) Muscle attaching to the cuticle, longitudinal section. Both (E, F), first abdominal segment of *A. dispar*. (G) Cells of a transporting epithelium under a (specialized) cuticle. Transporting epithelium in the collophore, *O. cincta*. (H) Cell neighboring an internal cavity; charging does not affect imaging of ultrastructural details. Trunk, *O. cincta*. (I) Border (asterisk) of two different types of cuticle. Appendage on the first abdominal segment of *A. dispar*. (J) Cuticle with a pattern characteristic for a springtail body. Collophore, *O. cincta*. (K) Base of a sensilla. Trunk, *O. cincta*. bl, basal labyrinth; gc, Golgi complex; gl, gut lumen; ld, lipid droplet; mit, mitochondria; mv, microvilli; ne, nuclear envelope; nu, nucleus; sen, sensilla; yellow arrowhead marks charging. Sample numbers (Additional file 6): 1 (A, B, D, G, J), 2 (H, K), 4 (C), 5 (E, F, I). Scale bars: (A, B, H, J), 1 μm; (C-G, I, K), 5 μm.

**Additional file 8:** Sub-slice imaging - example 1. Two primary beam energies were used to scan layers 25 nm apart with 50 nm physical sectioning. Sample: *O. cincta*, sample 1 (Additional file 6).

**Additional file 9:** Sub-slice imaging – example 2. Volume reconstruction of muscle and adjacent tissue. As in Fig. 4B, D, sample 3 (Additional file 6), showing all 3 axes cross-sections and volume rendering. Data was acquired by a combination of physical (40 nm) and virtual (10 nm) slicing. The voxel size was 8 x 8 x 10 nm^3^ in a volume of 16 x 12 x 13 μm^3^.

**Additional file 10:** Sub-slice imaging – example 3. Volume reconstruction of gut tissue. As in Fig. 4C, E, sample 6 (Additional file 6), showing all 3 axes cross-sections and volume rendering. Data was acquired by a combination of physical (40 nm) and virtual (10 nm) slicing. The voxel size was isometric 10 x 10 x 10 nm^3^ in a volume of 23 x 13 x 12 μm^3^.

## Data Availability

The full data sets generated and analysed during the current study are not publicly available, because they are part of biology research projects. They are available from the authors on reasonable request.

## Abbreviations

BSE: Backscattered electrons
FIB-SEM: Focused ion beam scanning electron microscopy
MR: Minimal resin
SE: Secondary electrons
SBF-SEM: Serial block-face scanning electron microscopy
SEM: Scanning electron microscopy
