# Supplementary material for: Minimal resin embedding of SBF-SEM samples reduces charging and facilitates finding a surface-linked region of interest
Source: Front Zool. 2023 Aug 29;20:29. doi: 10.1186/s12983-023-00507-x (PMC10463905; doi:10.1186/s12983-023-00507-x)
Supplement: Supplementary file 1 — Additional file 1. Protocol for heavy metal staining and resin infiltration used for samples in this study. [file 12983_2023_507_MOESM1_ESM.pdf]

Modified protocol by Hua et al. (2015) used for samples in this study

We followed the protocol by Hua et al. (2015), which stems from the original OTO protocol for SBF-SEM described by Deerinck et al. (2010).

All steps were carried out at room temperature (RT) unless specified. Note that all incubations in osmium tetroxide were at RT. All washes were 3 times for 15 min at RT or the last one was overnight at 4°C (if the work needed to be interrupted).

Protocol variant 1:
(1) The samples were washed in washing solution (0.15 M cacodylate buffer with 2 mM calcium chloride) and postfixed with 2 % osmium tetroxide in water for 1.5 h and then in 2.5 % potassium ferrocyanide for 1.5 h;
(2) washed with water and incubated in 1 % aqueous solution of thiocarbohydrazide for 2 h;
(3) washed with water and incubated in 2 % osmium tetroxide in water for 4 h;
(4) washed with water and incubated in 1 % aqueous uranyl acetate for 2 h;
(5) washed with water and incubated in Walton's block (lead aspartate) for 2 h at 60°C;
(6) washed with water and dehydrated with increasing concentrations of acetone (30 %, 50 %, 70 %, 80 %, 90 %, 95 %, 100 %) for 15 min in each step;
(7) infiltrated with increasing concentrations of Hard Plus 812 resin in acetone (1:2, 1:1, 2:1) for 1-2 h in each step and incubated in pure resin in a vacuum desiccator overnight.

Protocol variants 2 and 3 were similar to Protocol variant 1 with the following differences.

Protocol variant 2:
(3) incubation in 2 % osmium tetroxide in water was for 2 h
(4) incubation in aqueous uranyl acetate was overnight at 4°C

Protocol variant 3:
(1) postfixation was with 4 % osmium tetroxide in 3 % potassium ferrocyanide for 2 h;
(2) washed with washing solution
(3) incubation in 2 % osmium tetroxide in water was for 2 h

Protocol variant 1 was used for *A. dispar* and *O. cincta*, protocol variant 2 for *C. franzi*, protocol variant 3 for *O. cincta*.

References:
Deerinck TJ, Bushong EA, Thor AK, Ellisman M, Thor C. NCMIR methods for 3D EM: a new protocol for preparation of biological specimens for serial block face scanning electron microscopy. 2010; e SBEM Protocol v7_01_10.

Hua Y, Laserstein P, Helmstaedter M. Large-volume en-bloc staining for electron microscopy-based connectomics. Nat Commun. 2015;6:7923.
