# Supplementary figures and images for: Minimal resin embedding of SBF-SEM samples reduces charging and facilitates finding a surface-linked region of interest
Source: Front Zool. 2023 Aug 29;20:29. doi: 10.1186/s12983-023-00507-x (PMC10463905; doi:10.1186/s12983-023-00507-x)

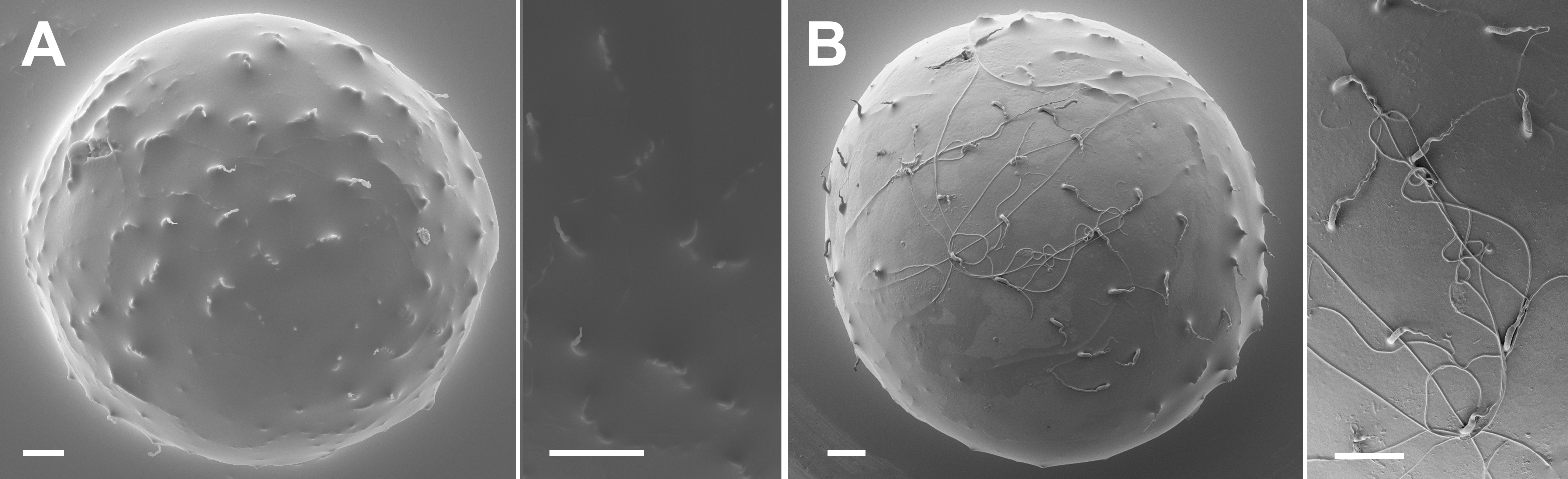

Supplement: Supplementary file 2 — Additional file 2: Comparison of details in surface ultrastructure between samples prepared without and with the optional acetone washing of external resin. (A) The acetone-washing step omitted, (B) included. Samples: roe of O. latipes. Scale bars: 100 μm. [file 12983_2023_507_MOESM2_ESM.tiff]

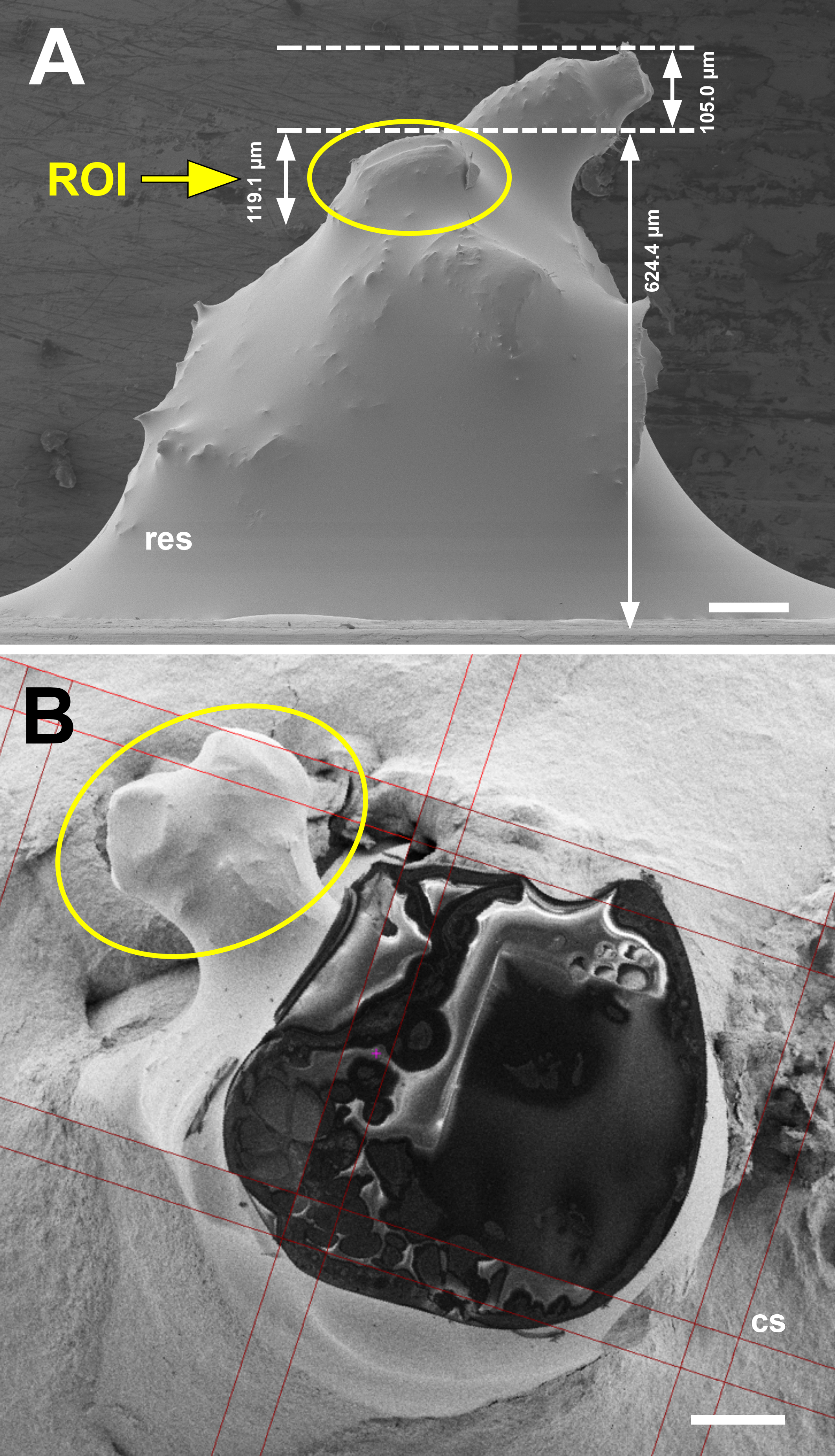

Supplement: Supplementary file 3 — Additional file 3: Additional illustrations of the preparation of MR embedded samples for SBF-SEM imaging. (A) Measuring the sample for precise trimming. External resin on this sample was removed by draining on absorbent paper without the acetone step, attached to the pin only with resin (without colloidal silver). Note that a structure (leg stump) is protruding slightly above the level of the ROI, which enables knife alignment. SBF-SEM images from this sample are shown in Fig. 3B and Fig. 4A. (B) Navigation to the ROI in the SBF-SEM microscope. Images in (A) and (B) were obtained using SE. cs, colloidal silver; res, resin; ROIs are encircled. Samples: O. cincta. Scale bars: 100 μm. [file 12983_2023_507_MOESM3_ESM.tiff]

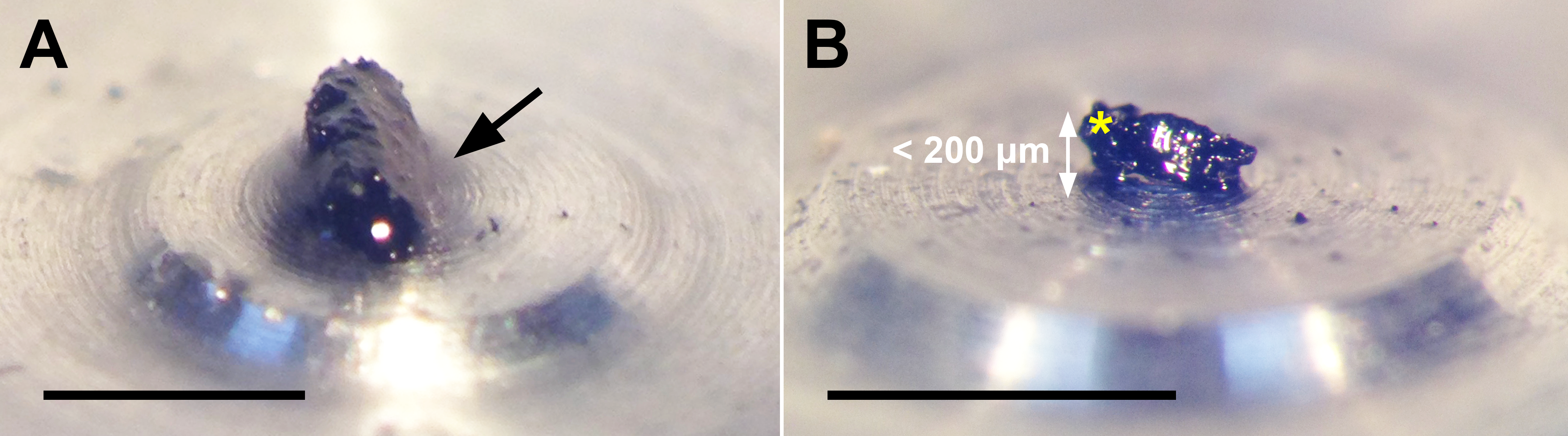

Supplement: Supplementary file 4 — Additional file 4: Incorrectly embedded samples. (A) Resin drop on the pin to attach the sample was too large. Resin rose up to the object (arrow). (B) Sample was very small, but mounted directly on the pin without the supporting pillar. Sectioning the ROI (asterisk) would take place too close to the pin (< ~200 μm). Samples: C. franzi (A) and A. dispar (B). Scale bars: 1 mm. [file 12983_2023_507_MOESM4_ESM.tiff]

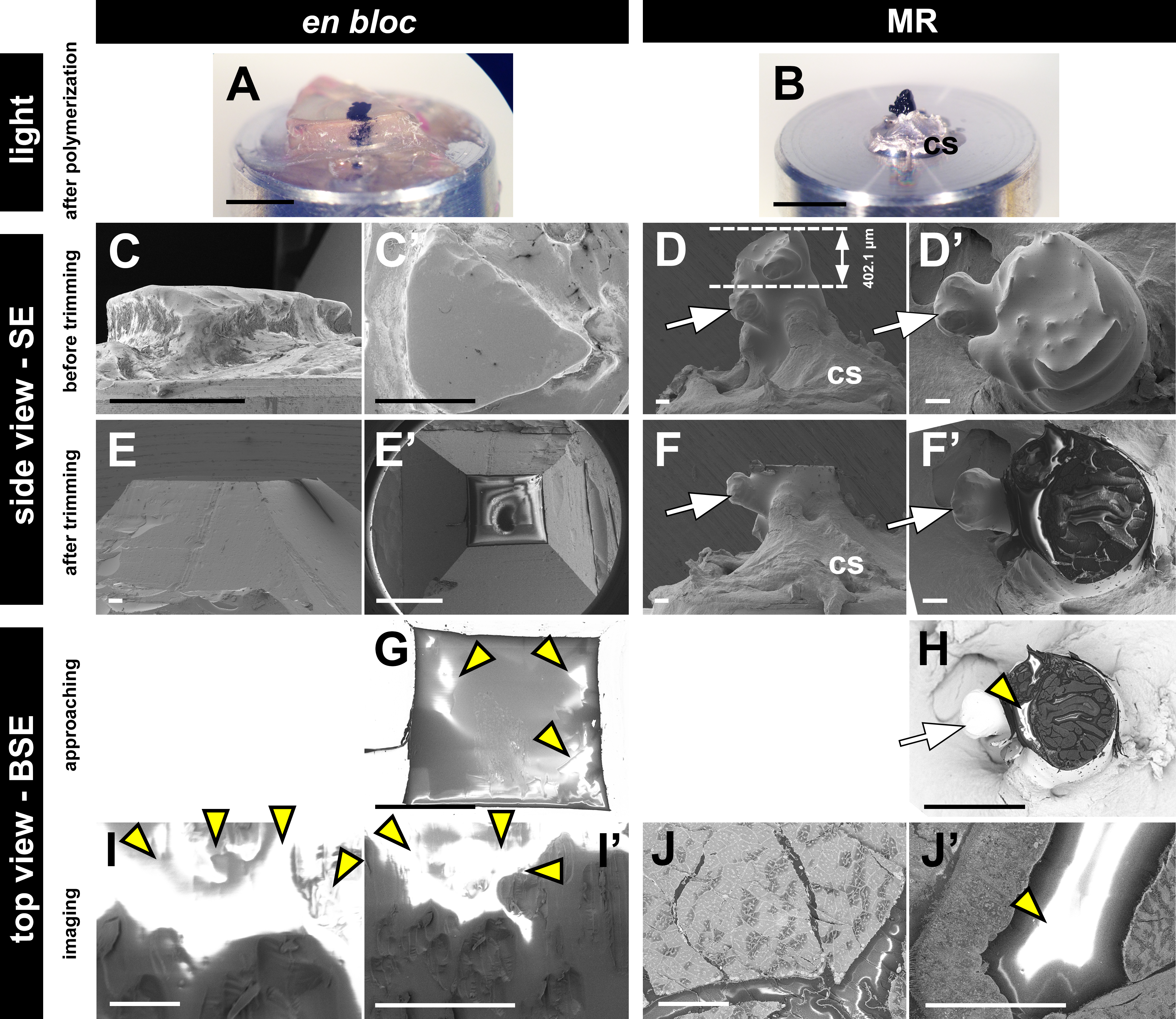

Supplement: Supplementary file 5 — Additional file 5: Trimming, approaching and sensitivity to charging in additional en bloc and MR embedded samples. (A, B) Polymerized samples on pins before they were gold coated. Colloidal silver was applied around the base of the sample in (B). (C-F’) Samples visualized in the classical SEM mode using SE. (C, E, D, F) are side views, (C’, E’, D’, F’) are top views. (C-D’) Measuring the length of the material above the ROI that has to be trimmed off. These values were only estimated in the en bloc embedded sample (C). (E-F’) Trimmed samples. A classical pyramid was made in (E, E’). (G-J’) Top views of samples visualized in the SBF-SEM mode using BSE. (G, H) Approaching. The ROI is easily localized and navigation to it is uncomplicated in the MR embedded sample (H). In (G) it is not clear how distant the ROI is from the top of the pyramid. Charging (yellow arrowhead) is visible in the resin surrounding the en bloc embedded sample (G). (I-J’) Images of embedded tissue after a few sections were cut, both samples in high vacuum. Minimal charging appears in the empty resin inside the MR embedded sample, which does not obscure details in the cells. Massive charging, which spreads into the tissue, appears in the en bloc embedded sample (I, I’) and completely disrupts imaging. (I, J) are at lower and (I’, J’) at higher magnification. cs, colloidal silver; white arrows mark the ROI; yellow arrowheads mark charging. Samples: O. cincta. Scale bars: (A, B), 2 mm; (C, C’), 2 mm; (E’), 1mm; (D, D’, E, F, F’), 100 μm ; (G, H), 500 μm; (I, J), 10 μm ; (I’, J’), 50 μm . [file 12983_2023_507_MOESM5_ESM.tiff]

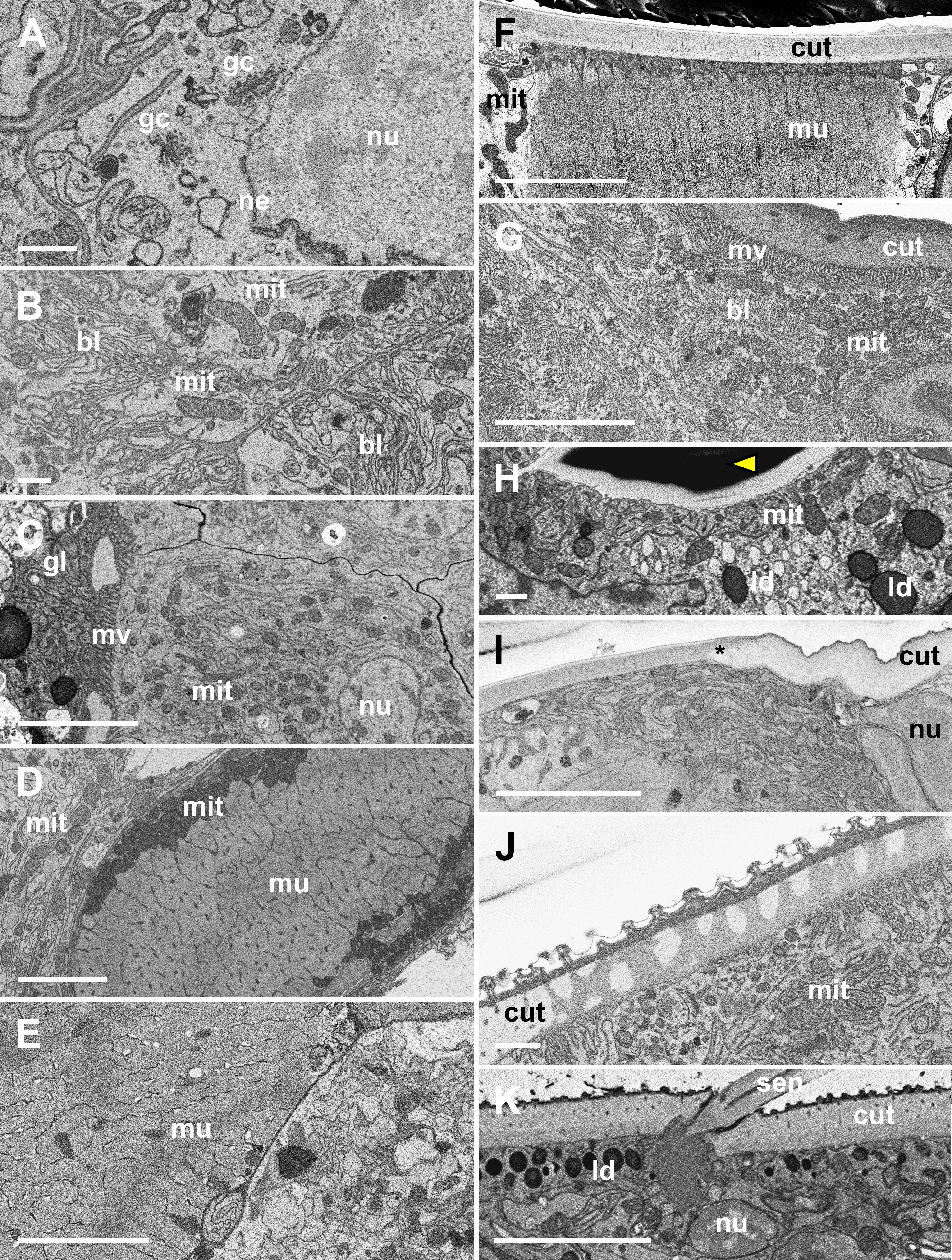

Supplement: Supplementary file 7 — Additional file 7: Ultrastructural details observed in SBF-SEM images of MR embedded samples. (A) Golgi complexes in the vicinity of the nucleus. (B) Mitochondria and the basal labyrinth (infolding of plasma membrane). Both (A, B), transporting epithelium in the collophore of O. cincta. (C) Gut cell, O. cincta. (D) Muscle, cross section. Collophore, O. cincta. (E) Muscle, cross section. (F) Muscle attaching to the cuticle, longitudinal section. Both (E, F), first abdominal segment of A. dispar. (G) Cells of a transporting epithelium under a (specialized) cuticle. Transporting epithelium in the collophore, O. cincta. (H) Cell neighboring an internal cavity; charging does not affect imaging of ultrastructural details. Trunk, O. cincta. (I) Border (asterisk) of two different types of cuticle. Appendage on the first abdominal segment of A. dispar. (J) Cuticle with a pattern characteristic for a springtail body. Collophore, O. cincta. (K) Base of a sensilla. Trunk, O. cincta. bl, basal labyrinth; gc, Golgi complex; gl, gut lumen; ld, lipid droplet; mit, mitochondria; mv, microvilli; ne, nuclear envelope; nu, nucleus; sen, sensilla; yellow arrowhead marks charging. Sample numbers (Additional file 6): 1 (A, B, D, G, J), 2 (H, K), 4 (C), 5 (E, F, I). Scale bars: (A, B, H, J), 1 μm; (C-G, I, K), 5 μm. [file 12983_2023_507_MOESM7_ESM.jpg]
